# Bio-corrosion impacts on mechanical integrity of ZM21 Mg for orthopaedic implant application processed by equal channel angular pressing

**DOI:** 10.1007/s10856-021-06535-5

**Published:** 2021-06-12

**Authors:** S. Prithivirajan, Mayur Bapu Nyahale, Gajanan M. Naik, S. Narendranath, Ashwini Prabhu, P. D. Rekha

**Affiliations:** 1 Corrosion Engineering Lab, Department of Mechanical Engineering, National Institute of Technology Karnataka, Surathkal, Srinivasanagar, Mangalore, Karnataka India; 2 Department of Mechanical Engineering, Mangalore Institute of Technology and Engineering, Moodbidri, Mangalore, Karnataka India; 3 Yenepoya Research Centre, Yenepoya Medical College, Yenepoya (Deemed to be University), Deralakatte, Mangalore, Karnataka India

## Abstract

The mechanical integrity of rolled ZM21 Mg was improved by equal channel angular pressing (ECAP) to function as a potential biodegradable bone screw implant. Electron backscattered diffraction (EBSD) revealed deformed grains of 45 µm observed in rolled ZM21 Mg. They were transformed to equiaxed fine grains of 5.4 µm after 4^th^ pass ECAP. The yield strength of rolled and ECAPed ZM21 Mg alloys were comparable. In contrast, 4^th^ pass ZM21 Mg exhibited relatively higher elongation when compared to rolled sample. The mechanical properties of rolled and ECAPed ZM21 Mg were dependant on both grain refinement and crystallographic texture. The rolled and 4^th^ pass ECAPed tensile samples exhibited nonlinear deterioration of mechanical properties when tested after 7, 14, 21 and 28 days immersion in Hank’s solution. The evaluation signifies that regardless their processing condition, ZM21 Mg alloys are suitable for surgical areas that requires high mechanical strength. In addition, the 4^th^ pass ECAP samples were viable to MG-63 cells proving themselves to be promising candidates for future in vivo studies.

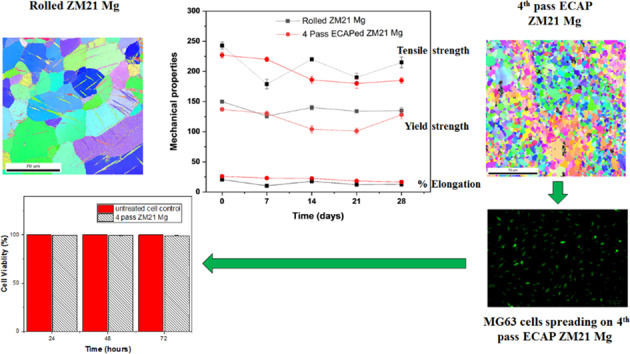

## Introduction

Biodegradable implants degrade in the human body and the degraded products are being consumed or excreted. Biodegradable implants should exhibit sufficient mechanical strength until healing is completed [1, 2]. Magnesium (Mg) and its alloys have been widely studied as degradable metallic biomaterials due to their degradability and the superior combination of strength and ductility over polymers and permanent implants. Mg alloys are potentially used as bone implants and stents due to their low density, inherent biocompatibility, and adequate mechanical properties [3, 4]. The elastic modulus of Mg alloys (40–45 GPa) is closer to that of human bones (10–40 GPa) than other commonly used implant materials [5]. As a result, the stress-shielding phenomena caused by current metallic implants made of stainless steel or Ti alloy can be minimised. As degradable materials, they will not remain in the body as permanent implants and will not require a second surgical operation after healing [6]. In spite of the numerous advantages, the use of Mg as a biodegradable implant has been restricted because of some limitations such as (i) relatively lower corrosion resistance (ii) hydrogen evolution [7–9]. In order to enhance the longevity of Mg implants, there is pressing need of a process that can simultaneously enhance mechanical properties as well as corrosion resistance [10, 11]. Researchers have chosen different methods viz., alloying [12], heat treatment [13], coatings [14], plastic deformation [15], and severe plastic deformation process to enhance the bio-corrosion resistance of magnesium alloys. Among versatile techniques available ECAP is most preferred. This is because it has the dual advantage of improving the bio-corrosion resistance and mechanical integrity of Mg alloys. The mechanical properties and biodegradation behaviour of ZM21 and ZK60 Mg alloys were improved by ECAP [16]. The compressive strength of ZK60 ECAPed samples enhanced compared to that of as received sample after immersion in PBS for 96 h [17]. The degradation behaviour of ZM21 Mg in Ringers solution upto 96 h using electrochemical impedance spectroscopy was evaluated [18]. However, these short time periods are not sufficient in evaluating the long term mechanical integrity of magnesium implants. From the literature, it is apparent that the application of magnesium implants in various surgical areas is being rediscovered. Meanwhile, there are few studies that reported the bio-mechanical properties of magnesium implants especially loss of mechanical integrity. Even though, the biomechanical properties of Mg alloys after degradation is reported [19–21]. From accessible literature there is no much focus on the technique to improve the bio-mechanical integrity of Mg alloys. Our previous studies improved the mechanical properties and corrosion behaviour of Mg alloys by ECAP [22–27]. The objective of the current study is to improve the biomechanical properties of ZM21 Mg alloy by ECAP for orthopaedic implant application.

## Materials and methods

### Materials and equal channel angular pressing

ZM21 Mg in rolled condition acquired from Exclusive Magnesium private limited, Hyderabad, India was used for the experiments. The composition of ZM21 Mg alloy include Zn-1.8, Mn-0.7, Fe-0.02, Ni-0.02 (Wt. %) and balance Mg. Equal channel angular pressing was carried on rolled ZM21 Mg samples. The samples were machined to 15.5 mm diameter and 95 mm length prior to severe plastic deformation by ECAP. ECAP die parameters were set to 110° die angle (ɸ), curvature of outer arc (ψ) 30°. The machined ZM21 Mg samples were lubricated with molybdenum disulphide was introduced in the input channel of ECAP die. The die was insulated with a cover of glass wool. The heating coils were turned on until temperature of the die reaches 220 °C. The sample was pressed by a plunger using universal testing machine while holding the ECAP die temperature at 220 °C. For the next pass, the sample was again machined to same 15.5 mm diameter but it was rotated 90° anticlockwise direction before placing in die channel following route Bc. ECAP was done on rolled ZM21 Mg sample up to 4 passes. Digital photograph of rolled and ECAPed samples are represented in the supplementary Fig. S1.

### Microstructural analysis

Electron back scattered diffraction (EBSD) was carried out on rolled, 1^st^ to 4^th^ pass ECAPed ZM21 Mg samples using FEG SEM (National Facility of OIM and Texture, IIT Bombay, India). Prior to EBSD, the samples were cut along extrusion direction, electro-polished and ion milled. Neighbour pattern averaging and re-indexing (NPAR) was also done to improve the success rate of indexing. The steps followed in the reference [28] was carried out. Pole figures, inverse pole figures and schmid factor data was generated from TSL OIM software version 8 for all ZM21 Mg samples.

### Mechanical characterisation

Tensile test was carried out on all the ZM21 Mg alloy samples machined according to ASTM E8M standards along extrusion direction. Sample dimension included gauge length of 20 mm and gauge diameter of 4 mm. The tests were conducted with horizontal table top electronic tensometer attached with DC servomotor. The crosshead speed was maintained at 0.5 mm/min. Tensile tests were triplicated to gain confidence in measurements.

### Corrosion behaviour

ACM Gill AC electrochemical corrosion setup was used to perform corrosion studies on rolled and ECAPed ZM21 Mg alloys. Each sample was cut to 2 mm thickness along the extruded direction and abraded with #2000 grit emery papers. Mirror like surface finish was obtained by polishing using diamond paste of 0.25 µm. Hank’s solution was used as testing medium. ZM21 Mg alloy sample served as working electrode, saturated calomel as reference electrode and graphite rod as counter electrode. Electrochemical impedance spectroscopy (EIS) test was carried out in the frequency range of 10000–0.01 Hz with an amplitude of 10 mV with respect to open circuit potential (OCP). Cyclic sweep tests were conducted by sweeping through a potential from −250 mV to +250 mV and scan rate of 1 mV/s. The Nyquist plots were fitted using V4 analysis software. Corrosion parameters such as corrosion potential (E_corr_) and corrosion current density (i_corr_) were calculated by Tafel extrapolation of cathodic slope. All tests were conducted at 37 °C in Hank’s solution and were repeated three times to gain confidence in measurements.

### In vitro loss of mechanical integrity

Rolled and 4^th^ pass samples were machined according to ASTM E8-M standards. All the samples were placed in incubator maintained at 37 °C. Each sample was immersed in 30 ml Hank’s solution and was also replaced every three days. The samples immersed in Hank’s solution for various duration of 7, 14, 21 and 28 days were removed. They were then subjected to tensile testing. Tensile testing was carried out (triplicated, *n* = 3) on rolled and 4^th^ pass ECAPed ZM21 Mg alloy at crosshead speed of 0.5 mm/min.

### Cytotoxicity evaluation

#### Cell and culture conditions

Human osteoblast-like cells (MG63) were used for the evaluation of cytotoxicity. They were cultured in Dulbecco’s modified eagle’s medium (DMEM) supplemented with 10% foetal bovine serum (FBS) and 1% antibiotic–antimycotic solution. Cells were maintained at 37 °C and 5% CO_2_ in a humidified atmosphere throughout the experiments.

#### Assessment of cytotoxicity by MTT assay

Cytotoxicity of test materials was assessed using methyl thiazolyl tetrazolium (MTT) assay [29]. Cells were seeded onto 24 well microtiter plates at a seeding density of 25000 cells/well. After adherence, they were treated with the 4^th^ pass ECAPed ZM21 for different time points viz., 24, 48, and 72 h. After the stipulated treatment period, MTT reagent was added to the wells and incubated at 37 °C for 4 h. Formazan crystals formed were solubilized using dimethyl sulfoxide (DMSO) and absorbance was recorded at 570 nm using multimode microplate reader (Fluostar Omega, BMG Labtech). Percentage cytotoxicity of the test compound was calculated with respect to untreated cell control.

## Results and discussion

### Microstructure of ZM21 Mg

**Fig. 1:**
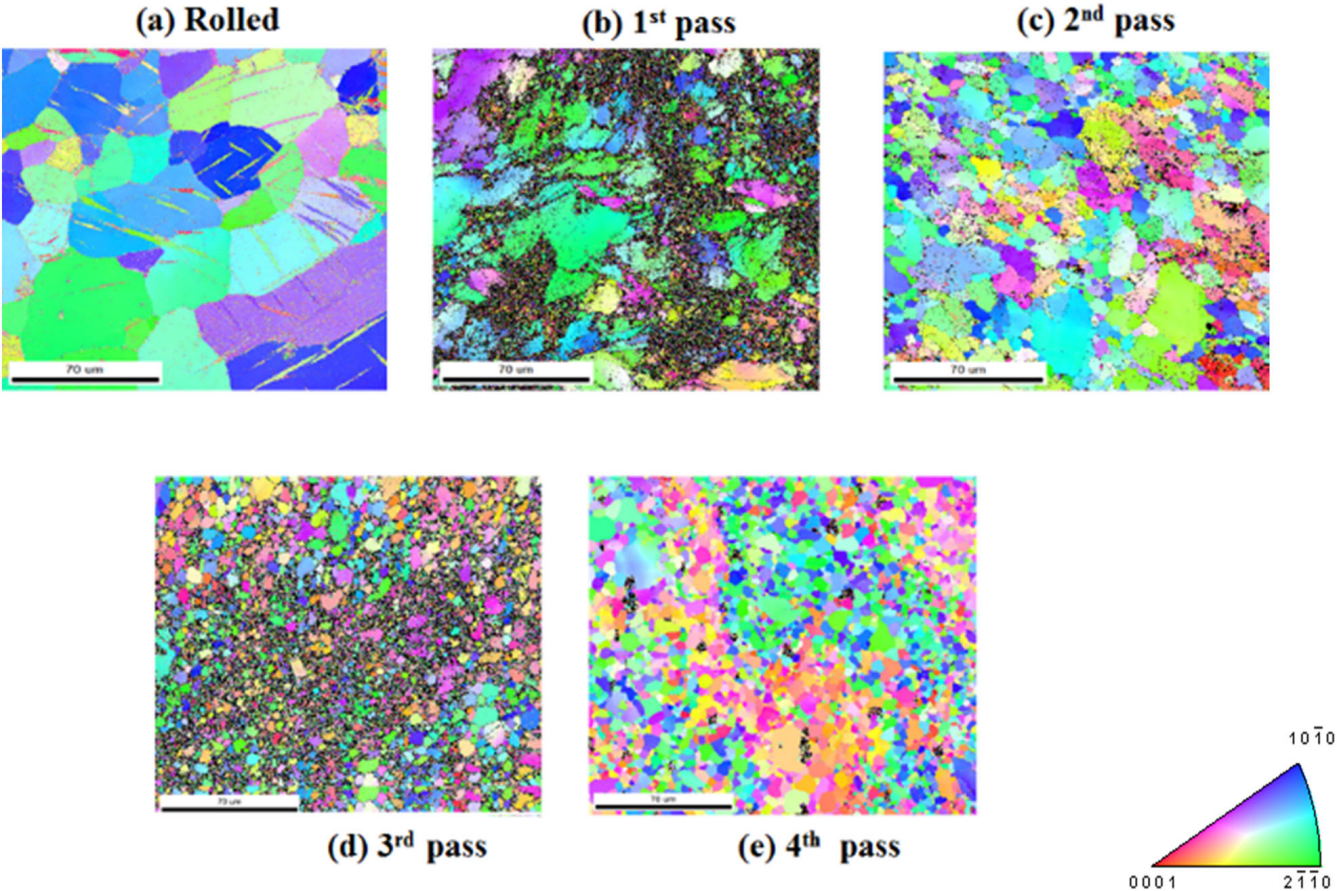
EBSD Micrographs of ZM21 Mg **a** Rolled **b** 1^st^ pass **c** 2^nd^ pass **d** 3^rd^ pass **e** 4^th^ pass

The electron backscattered diffraction (EBSD) micrographs of ZM21 Mg is depicted in Fig. 1(a–e). ZM21 Mg exhibited deformed grains with average size of 45 µm as a result of rolling. The formation of mechanical twins is evident from EBSD micrograph depicted in Fig. 1(a). It is well established that plastic deformation in metallic materials occurs by slip and twinning [30]. Hence the occurrence of twinning is observed in the present study after rolling of ZM21 Mg alloy. The dominant role of twinning during rolling process is attributed especially to limited number of slip systems in Magnesium [30]. Recent studies also reported the formation of twins in ZX11 Mg alloy when processed by twin roll casting technology [20]. Figure 1 (b) represents the EBSD orientation map of sample after 1^st^ pass of ECAP. A bimodal distribution was observed with mean size of 0.56 µm (DRXed grains) and 39 µm at area fraction of 0.109 and 0.164 respectively measured from EBSD. It is also interesting to note that mechanical twins disappeared after 1^st^ pass of ECAP. This is probably due heating samples from room temperature to 225 °C prior to ECAP. Similar observations were reported in ZX11 Mg alloys [20]. Also, in contrast to rolling where deformation is dominated by twinning, the mechanism of severe plastic deformation occurs by slip and simple shear during ECAP. Even after completion of 2^nd^ pass ECAP, bimodal grain distribution prevailed with grains of 6 µm and 36 µm diameter. Bimodal grains are inherent during ECAP and were also observed by various researchers [16, 31]. The 3^rd^ pass of ECAP exhibited average grain diameter of 5 µm. Finally, fine grains of 5.4 µm with equi-axed grain structure was achieved after 4^th^ pass of ECAP. The minimal grain growth experienced from 3^rd^ pass (5 µm) to 4^th^ pass (5.4 µm) is due to the instability of fine grains at 225 °C (refer Fig. 1 (d) and (e)). The grain refinement obtained in this study is in complete agreement with models proposed elsewhere [32].

### Evolution of texture element during ECAP

**Fig. 2:**
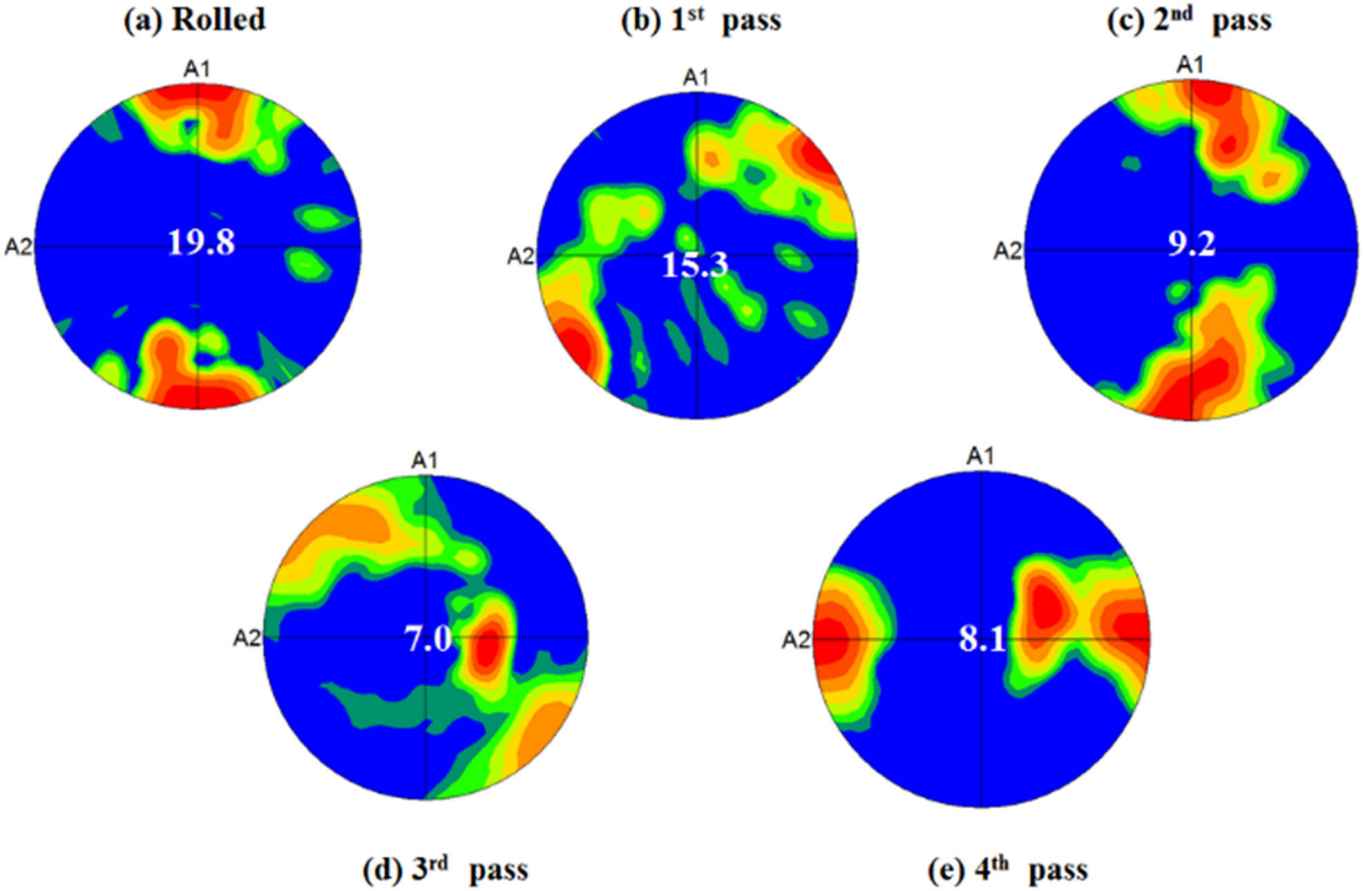
Basal pole figures of ZM21 Mg **a** Rolled **b** 1^st^ pass **c** 2^nd^ pass **d** 3^rd^ pass **e** 4^th^ pass A1: Extrusion Direction (RD) A2: Transverse Direction (TD)

The basal pole figures of ZM21 Mg alloy in rolled and ECAPed condition is represented in the Fig. 2 (a–e). The rolled ZM21 Mg exhibited texture along rolling direction (RD) with maximum intensity of 19.8 as mentioned in Fig. 2(a). After completion of 1^st^ pass the texture element shifted ~45° to RD/TD (transverse direction) along with a relative decrease in texture intensity. The shift in position of maximum texture intensity is related to rotation of ZM21 Mg sample by 90° in anticlockwise direction between each pass of ECAP. The 2^nd^ pass of ECAP also exhibited texture along RD comparable to rolled ZM21 Mg but the texture intensity decreased to a value of 9.2. However, 4^th^ pass ECAP resulted in shifting the texture to a location away from RD and TD. It is also interesting to observe relative decrease in texture intensity from 19.8 in rolled condition to 15.3, 9.2 and 7.0 after 1^st^, 2^nd^, and 3^rd^ pass of ECAP respectively. In contrast, a slight increase in pole intensity of 8.1 was observed at the completion of 4^th^ pass. Moreover, the 4^th^ pass ZM21 Mg exhibited a texture parallel to RD. This decrease in texture intensity from rolled condition to 3^rd^ pass ECAP pass signifies weakening of initial texture element. While, the slight increase in texture intensity indicates the formation of new texture element. Generally, rolling and extrusion results in formation of strong texture element. It is well known that extruded magnesium alloys exhibits fibre texture in contrast to random texture observed in cast Mg alloys [16, 31]. However, after ECAP the initial texture of wrought and cast magnesium alloys is disintegrated eventually leading to weakening and formation of new texture element [16, 28, 31, 33]. The present study also showed similar phenomenon which is apparent from Fig. 2(a–e).

### Combined influence of grain refinement and texture on mechanical properties of ZM21 Mg alloy

The mechanical properties of rolled and ECAPed Mg is tabulated in Table 1. Rolled ZM21 Mg sample exhibited yield strength of 150 MPa and elongation of 20%. The yield strength decreased relatively to 136 MPa after completion of 1^st^ pass ECAP. Despite the decrease in grain size from 45 µm in rolled condition to 18.4 µm during 1^st^ pass of ECAP there was a relative drop in yield strength. This is due the fact that the basal texture of 1^st^ pass ZM21 Mg represented in Fig. 2(b) was aligned at ~45° to RD/TD favouring the occurrence of basal slip. This phenomenon in turn lowered the stress necessary for yielding. In contrast, 2^nd^ pass of ECAP evinced relatively higher value of yield strength 154 MPa compared to all the ZM21 Mg samples. This signifies that the effect of grain refinement dominated during 2^nd^ pass of ECAP despite the alignment of texture along RD. It is apparent from Fig. 1 (d) and (e) that successive ECAP 3^rd^ and 4^th^ passes respectively did not show remarkable grain refinement. Moreover, increment in ECAP passes leads to linear decrement in texture intensity from 19.8, 15.3, 9.2 in rolled, 1^st^ pass, 2^nd^ pass condition to 7.3 after 3^rd^ pass of ECAP. This is followed by relative increase in intensity of 8.1 as represented in Fig. 2. Thus, it can be concluded that during 3^rd^ and 4^th^ pass of ECAP basal texture dominated the effect of grain refinement. The 4^th^ pass of ECAP exhibited maximum texture parallel to RD. Such orientation did not lower the yield strength significantly. In summary, the yield strength of ZM21 Mg is influenced by both grain refinement and texture. It is also known that extruded Mg samples have typical fibre texture. However, equal channel angular pressing leads to texture softening ending up in formation of new texture element. [16, 17, 31, 34–36] Not much literature is available on texture evolution of ECAPed Mg alloys whose initial processing condition is rolling. However, in the present study pole figures (refer Fig. 2) generated from EBSD enlightens the impact of texture on mechanical properties of ZM21 Mg. In general, rolling or extrusion results in strong texture components leading to deterioration in percentage elongation. Hence, the percentage elongation of rolled ZM21 Mg was measured to be 20% and is found to be lower than all ECAPed samples as mentioned in Table 1. Schmid factor value for the occurrence of basal slip was found to be 0.22 in rolled condition and for 1^st^, 2^nd^, 3^rd^, and 4^th^ pass ECAP it is 0.25, 0.32, 0.33, and 0.35 respectively. Thus, ECAP enhanced the percentage of elongation due to increase in Schmid factor values. Recent reports on ECAP of ZK60, ZM21, AZ61, LAE442, AE21 and AE42 magnesium alloys also showed similar increasing trends in percentage elongation [16, 28, 31, 37, 38].

**Table 1:** Mechanical properties of ZM21 Mg

ECAP Pass	Grain Size (µm)	Yield Strength (MPa)	Ultimate Tensile Strength (MPa)	Elongation%
0	45	150 ± 5	243 ± 6	20 ± 2
1	18.4	136 ± 6	228 ± 7	21 ± 1
2	10.9	154 ± 3	215 ± 4	22 ± 2
3	5.0	128 ± 4	212 ± 5	23 ± 2
4	5.4	137 ± 5	227 ± 6	27 ± 1

### Short term corrosion behaviour of ZM21 Mg

#### Electrochemical corrosion of ZM21 Mg

**Fig. 3:**
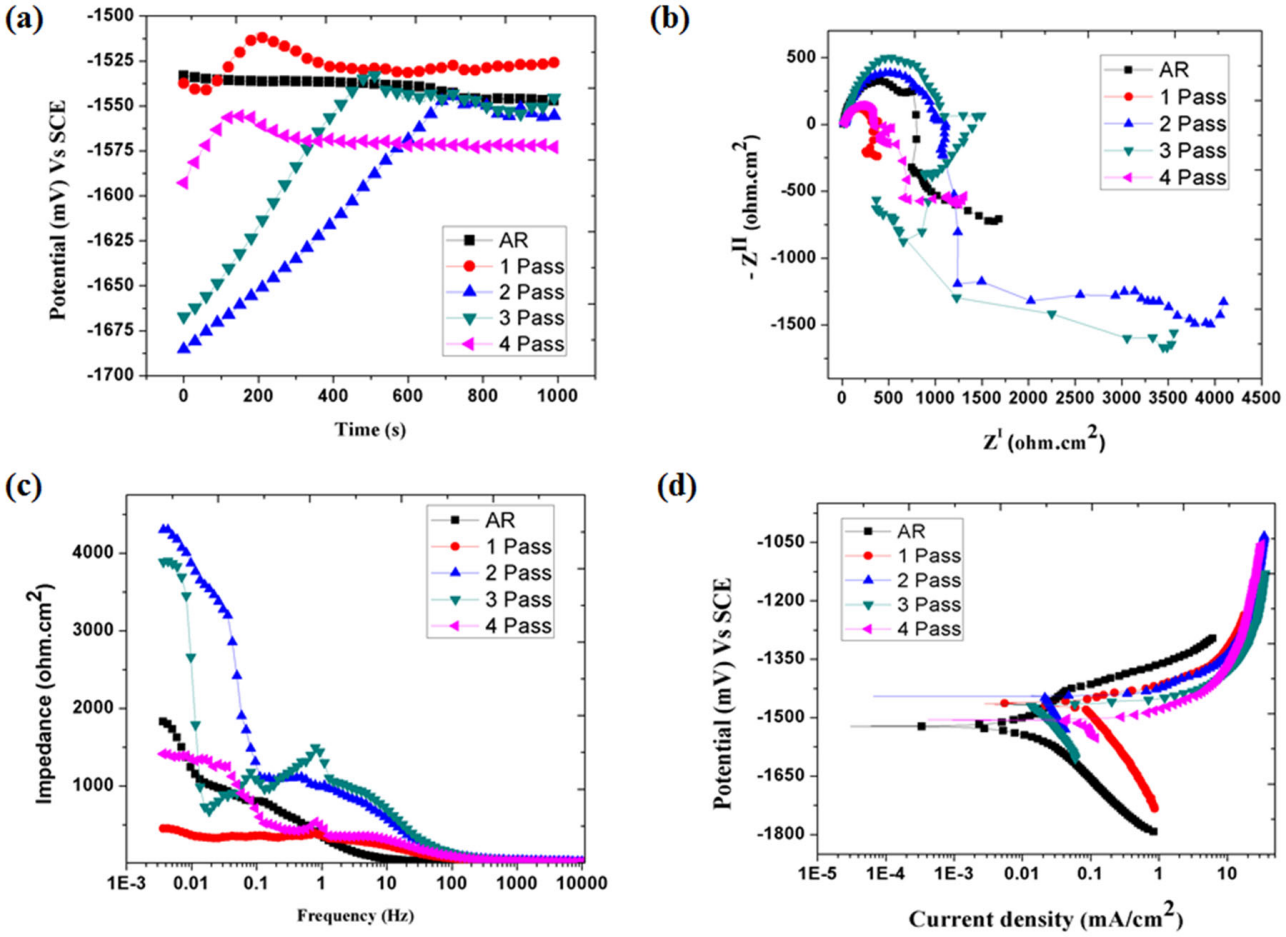
Short term corrosion behaviour of ZM21 Mg (a) Open circuit potential (b) Nyquist plot (c) Bode Impedance Plot (d) Potentiodynamic polarisation curve

The corrosion behaviour of rolled and ECAPed ZM21 Mg is analysed using electrochemical studies and is represented in the Fig. 3 (a–d). The open circuit potential of rolled and ECAPed samples is depicted in the Fig. 3(a). The rolled sample exhibited linear decrease in potential with respect to time period of 1000 seconds. This is related to the presence of defects such as mechanical twins observed in EBSD micrograph shown in Fig. 1(a). In contrast, all the ECAPed samples exhibited initial linear increase and then attained equilibrium after 800 seconds. This linear increase in electrode potential of ECAPed samples is attributed to fine distribution of secondary phase. ZM21Mg samples in increasing order of corrosion resistance is ranked as follows 1^st^ pass >3^rd^ pass > rolled >2^nd^ pass > 4^th^ pass. However, open circuit potential curves do not give information about properties of surface film. Hence, electrochemical impedance spectroscopy was carried out on all ZM21 Mg samples and the results are presented in the Fig. 3 (b). All the ZM21 Mg samples exhibited high and medium frequency capacitive loops followed by a low frequency inductive loop which is typical characteristic of magnesium and its alloys. Generally, the high frequency, medium frequency capacitive loop and low frequency inductive loop are related to charge transfer resistance (Rct), robustness of surface film and adsorption of species respectively. The diameter of capacitive loop is proportionate to corrosion resistance of the material. Hence, the order of corrosion resistance based on charge transfer resistance is ranked as follows 3^rd^ Pass >2^nd^ pass > rolled >4^th^ pass >1^st^ pass. However, impedance (Z) is a function of charge transfer resistance, solution resistance and double layer capacitance [39]. The rank of corrosion resistance based on value of impedance is 2^nd^ pass >3^rd^ pass > rolled >4^th^ pass >1^st^ pass. It is also interesting to note that the corrosion resistance neither increased nor decreased in chronological order with increment in ECAP passes. To understand the reason behind the trend, the role of secondary phase particles, crystallographic orientation and ions present in corrosive medium are taken into consideration.

#### Role of secondary phases on ZM21 Mg corrosion

**Fig. 4:**
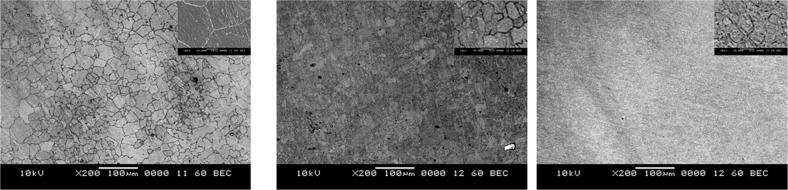
scanning electron micrographs of ZM21 Mg **a** Rolled **b** 2^nd^ pass **c** 4^th^ pass

From EBSD figures represented in the Fig. 1(a–e), it is clear that the secondary phase particles are not clearly indexed. Hence, the SEM images of rolled, 2^nd^ pass and 4^th^ pass ZM21 Mg samples are depicted in the Fig. 4(a–c). It is apparent from Fig. 4 (a) that the secondary phase particles are decorated along the grain boundaries in rolled ZM21 Mg. After, ECAP they are fragmented into finer particles and distributed uniformly which is apparent from inset of Fig. 4(b, c). In general, the anodic partial reaction occurs by the dissolution of magnesium and release of an electron as mentioned in Eq. (2). This electron is taken up at MgZn_2_ cathodes resulting in hydrogen evolution as shown in Eq. (1). In addition to electrochemical mode of corrosion, dissolution of magnesium occurs by chemical reaction which is referred as anodic hydrogen evolution mentioned in Eq. (3). The overall reaction represented in the Eq. (4) includes corrosion of magnesium by electrochemical and chemical mode. In addition, corrosion product formation is mentioned in Eq. (5). The potentiodynamic polarisation plots of ZM21 Mg represented in Fig. 3(d) indicated that rolled ZM21 Mg exhibited Tafel behaviour with equal contribution from cathodic and anodic reaction. This is due to relatively larger size (~1.5 µm) of secondary phase in rolled ZM21 Mg playing major role in driving the cathodic reaction. In contrast, all the ECAPed samples deviated from Tafel behaviour with anodic reaction and chemical corrosion of ZM21 Mg dominating the cathodic reaction. This is because during equal channel angular pressing, the secondary phase particles (cathodes) are fragmented and distributed into finer particles (~0.4 µm) and anodic hydrogen evolution occurs. Similar kind of potentiodynamic polarisation (PDP) curves were reported in ZM21 Mg when phosphate buffer solution was used as a testing medium [40]. The parameters deciding the corrosion behaviour of rolled and ECAPed ZM21 Mg alloys are presented in Table 2. The corrosion rate of all the samples under study are comparable due to extra resistance occurring between magnesium and secondary phases. Also, micro-galvanic corrosion is suppressed due to less aggressive nature of Hank’s solution [41]. The corrosion resistance of ZM21 Mg samples based on potentiodynamic polarisation plots is given as rolled >2^nd^ pass >3^rd^ pass >4^th^ pass >1^st^ pass. The results from electrochemical impedance spectroscopy (EIS) and potentiodynamic polarisation (PDP) did not show similar trend, because EIS is nondestructive while PDP is destructive. In addition, EIS considers the robustness of surface film while PDP is based on anodic, cathodic partial reaction, and chemical reaction of magnesium alloys.

1 $$2{\mathrm{H}}^ + + 2{\mathrm{e}} \to {\mathrm{H}}_2\left( {{\mathrm{cathodic}}\,{\mathrm{partial}}\,{\mathrm{reaction}}} \right)$$

2 $$2{\mathrm{Mg}} \to 2{\mathrm{Mg}}^ + + {\mathrm{e }}\left( {{\mathrm{anodic}}\,{\mathrm{partial}}\,{\mathrm{reaction}}} \right)$$

3 $$2{\mathrm{Mg}}^ + + 2{\mathrm{H}}_2{\mathrm{O}} \to 2{\mathrm{Mg}}^{2 + } + 2{\mathrm{OH}}^ - + {\mathrm{H}}_2\left( {{\mathrm{chemical}}\,{\mathrm{reaction}}} \right)$$

4 $$2{\mathrm{Mg}} + 2{\mathrm{H}}^ + + 2{\mathrm{H}}_2{\mathrm{O}} \to 2{\mathrm{Mg}}^{2 + } + 2{\mathrm{OH}}^ - + 2{\mathrm{H}}_2\left( {{\mathrm{overall}}\,{\mathrm{reaction}}} \right)$$

5 $${\mathrm{Mg}}^{2 + } + 2{\mathrm{OH}}^ - \to {\mathrm{Mg}}\left( {{\mathrm{OH}}} \right)_2\left( {{\mathrm{product}}\,{\mathrm{formation}}} \right)$$

**Table 2:** Corrosion behaviour of ZM21 Mg

ECAP Pass	R_ct_ (Ω.cm^2^)	R_sol_ (Ω.cm^2^)	C_dl_ (F)	Z (Ω.cm^2^)	Potential (mV)	Current density (mA/ cm^2^)	Corrosion Rate (mm/yr)
0	762.6	15.34	4.154 E-04	1822	−1522	0.045	1.028
1	307.7	18.68	3.878 E-05	442	−1462	0.146	3.336
2	980.8	22.13	1.135 E-05	4334	−1446	0.047	1.078
3	1085	20	7.227 E-06	3902	−1468	0.056	1.279
4	331.3	21.50	1.492 E-04	1406	−1506	0.087	1.987

#### Influence of crystallographic orientation on ZM21 Mg corrosion

**Fig. 5:**
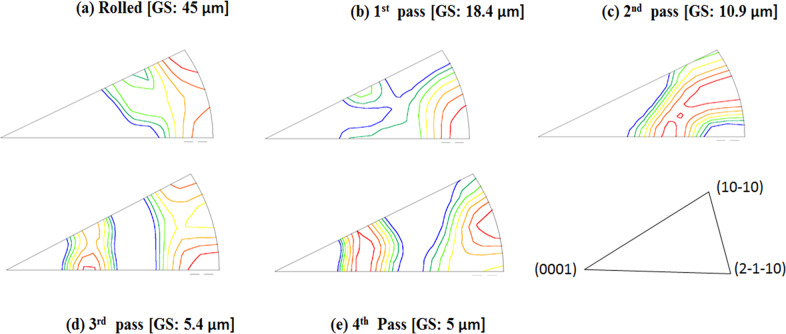
Inverse pole figures of ZM21 Mg **a** Rolled **b** 1^st^ pass **c** 2^nd^ pass **d** 3^rd^ pass **e** 4^th^ pass

To further enlighten the reason for such corrosion behaviour the crystallographic orientation of ZM21 Mg was also analysed. From Fig. 1(a) the following observations are made (i) the average grain size after rolling is 45 µm (ii) appearance of mechanical twins (iii) majority of the grains are oriented towards (10-10) and (2-1-10) prism planes which is also in agreement with Fig. 5(a). Increasing ECAP passes resulted in fragmentation of secondary phases which is observed from Fig. 4 (a–c). Figure 5 (a–e) represents the inverse pole figures (IPF) of rolled and ECAPed ZM21 Mg along with their grain size for easier comparison. It is apparent that the rolled, 1^st^ pass, 2^nd^ pass exhibited majority of orientation towards (10-10)/ (2-1-10) prism planes. In contrast, 3^rd^ pass and 4^th^ pass samples evinced majorly (10-10)/ (2-1-10) prism orientation along with (0001) minor basal orientation. The combination of basal and prism orientation also form a galvanic cell thereby relatively deteriorating the corrosion resistance [42]. Hence, the finer grain size along with the favourable orientation is expected to exhibit higher corrosion resistance. Recent studies on ECAPed Mg alloys revealed enhancement in corrosion resistance of ZE41, ZM21, AZ80 [16–18, 26, 27] on the other hand unfavourable effects was observed in AZ91D Mg alloy [43]. Jamesh et al [18]. reported that during corrosion behaviour evaluation of ZM21 Mg, significant change in Nyquist plot was observed after every 4 h duration starting from 0 to 92 h immersion. This clearly enlightens the fact that magnesium corrosion is dynamic in nature. This is the reason for the variation of trends in corrosion resistance obtained from EIS and PDP methods in the present study as depicted in the Table 2. However, the short term results evaluated from EIS and PDP is not enough for completely comprehend the corrosion behaviour of Mg implants. This is because when used as an implant they are subjected to long term corrosion in terms of months. Hence, in the current study further efforts have been made to assess long term mechanical integrity of rolled (G.S - 45 µm) and 4^th^ pass equiaxed (G.S - 5 µm) ZM21 Mg.

### Long term mechanical integrity ZM21 Mg

**Fig. 6:**
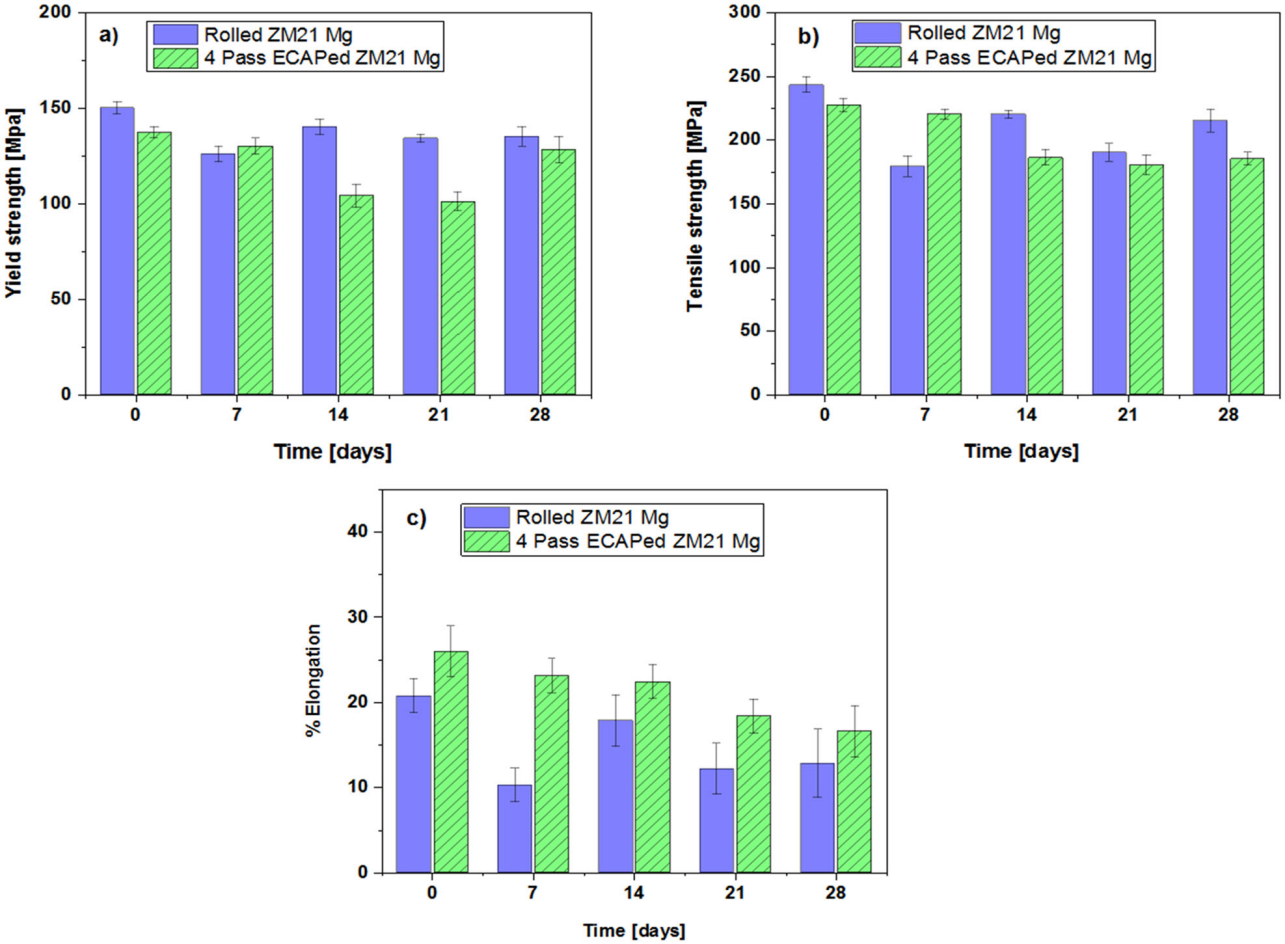
Mechanical properties of rolled and 4^th^ pass ECAPed ZM21 Mg **a** Yield strength **b** Tensile strength **c** % elongation after 28 days immersion in Hank’s solution maintained at 37 °C

The mechanical properties of all ZM21 Mg after immersion in Hank’s solution for 0, 7, 14, 21 and 28 days is presented in the Fig. 6 (a–c). Digital photograph of ZM21 Mg before and after 28 days are depicted in supplementary Fig. S2. From Fig. 6 (a–c) it is observed that the mechanical properties of all ZM21 Mg under study deteriorates with respect to time. This is corroborated to the degradation of ZM21 Mg leading to cross section reduction and possibly due to pit formation. Surprisingly, the mechanical properties did not decrease linearly with increase in days. ZX11 Mg was developed to function as a bone plate implant. After 28 days of immersion % elongation of both rolled and annealed ZX11 Mg alloy deteriorated to less than 4% elongation. This is probably due to relatively lower thickness of 1.8 mm ZX11 plate used [20]. They also reported a nonlinear trend in deterioration of mechanical properties with respect to time. However, the current study focusses on the bone screw application. It is noteworthy to mention the success of MAGNEZIX screws implanted for treatment of Hallux Valgus [44–47], lateral malleolar fracture [48], extra-articular and intra-articular fractures, humerus fracture [49], mandible fracture [50]. However, there are some reports where MAGNEZIX screws are not recommended due to bone cysts formation [51, 52]. The reason for such adverse effect may be due relatively lower elongation (>8%) and also the average time required for healing of scaphoid fracture is greater than 10 weeks. Hence, it is reasonable to conclude that these implants were successful in healing factures which can be healed within 10–12 weeks. Further fundamental research is necessary to enhance the biomechanical integrity and longevity of Mg alloys [53]. MAGNEZIX screws are processed by adapted casting, powder metallurgy and extrusion techniques [54]. Generally, extrusion of Mg alloys results in formation of fibre texture which is detrimental to ductility. In present study, the mechanical properties of ZM21 Mg in rolled condition (YS: 150 MPa, UTS: 243 MPa % El: 20) and 4^th^ pass ECAPed condition (YS: 137 MPa, UTS: 227 MPa: % El: 27) are comparable to that of MAGNEZIX implants. Equal channel angular pressing of ZM21 Mg resulted in the highest elongation of 27% after 4^th^ pass attributed to higher schmid factor for basal slip. After, 28 days immersion in Hank’s solution at 37 °C the % elongation of rolled and 4^th^ passed ZM21 were reported to be 12.82 % and 16.6 %. Hence, ECAP is a promising technique for enhancing mechanical integrity of Mg implants. The degradation rate of pure Mg and its alloys in in vitro conditions is higher than that experienced in in vivo [55]. It is noteworthy to mention that efforts have been made to bridge the gap between in vivo and in vitro testing. In addition, pure Mg, AZ31, Mg-0.8Ca, Mg-1Zn, Mg-1Mn and Mg-1.34Zn–Ca alloys exhibited relatively lower degradation rate in vivo compared to in vitro condition [56, 57]. Hence, it is reasonable to believe that ZM21 Mg alloys used in present also has possibilities to exhibit relatively lower degradation in in vivo studies. The extruded ZM11 Mg alloy implanted into femora of rabbits and rats exhibited good bioactivity during 4 weeks and 18 weeks of implantation respectively [58, 59]. Even though, some of the magnesium implants were successful in in vivo experiments on rats and rabbits. The results from this experiments cannot be used to predict their behaviour in human body. This is because the blood flow rate of rats and rabbits are 10 to 6 times lower than that measured in the human bone respectively. Also, fracture healing time of parts in lower limbs of humans is relatively greater than upper limb [53]. Hence, the future studies should further improve the longevity of implants so that they will be able to heal fractures at lower limb.

### Cell viability of ZM21 Mg

**Fig. 7:**
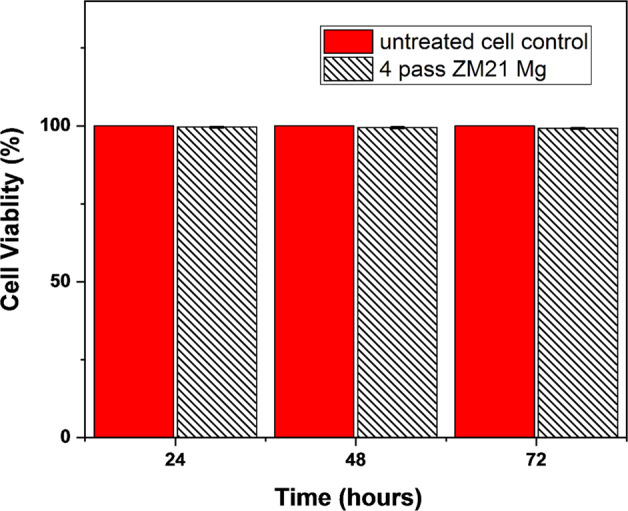
Cell viability of 4^th^ pass ECAP sample as a function of time

**Fig. 8:**
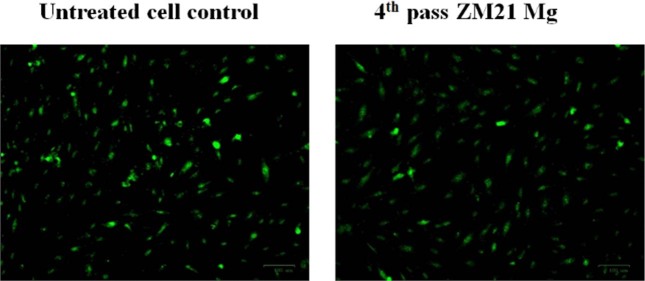
Live/dead staining of MG63 cells on 4^th^ pass ZM21 Mg

Cell viability of 4^th^ pass ECAPed ZM21 Mg samples are represented in Fig. 7. The cell viability % of 4^th^ pass sample were found to be 99.58 ± 0.15, 99.44 ± 0.17 and 99.21 ± 0.18 at 24, 48 and 72 hours respectively. Cytocompatibility is further verified by analysing the live/dead MG63 cells staining using the fluorescent images represented in the Fig. 8. The images evinced that when compared to untreated control relatively large number of living cells were found in 4^th^ pass ZM21 Mg. This proves good bio compatibility of 4^th^ pass ZM21 Mg after 72 hours of cell culture. It is observed that the cell viability % varied with increase in different time periods as depicted in Fig. 7. Similar results of cell spreading was obtained when ZX11 Mg was cultured in human primary osteoblasts [20, 60]. The variation in cell viability ratio and appearance of dead cells is due to degradation Mg corrosion. Witecka et al [43]. reported behaviour of ZM21 Mg in different simulated body fluids. They observed that pH of ZM21 Mg for a period of 24 hours was ~7 which is one of the reasons for increasing magnesium corrosion. It is reported during cytotoxicity testing of magnesium, there is a chance of corrosion products dominating the measurement process. Also, the disadvantage of using tetrazolium salts for cytotoxicity testing is that they are qualitative in nature [61]. So, in the present study cell attachment and spreading is also carried to conform the cell viability. Hence, it is reasonable to believe that the fine grains (refer Fig. 1(e)) and uniform distribution of secondary phases (refer Fig. 4(c)) of 4^th^ pass ECAP sample aided in cell adhesion and viability. So, both cell viability ratio and cell spreading indicated that 4^th^ pass ZM21 Mg is biocompatible in nature.

## Conclusion

In this study, mechanical integrity of ZM21 Mg is enhanced by equal channel angular pressing for orthopaedic application. The conclusions are discussed below.

1. By utilising 4-pass ECAP at 220 °C, the ECAPed sample exhibits a mean grain size of 5.4 µm with uniformly distributed MgZn_2_ secondary phase. The texture intensity of as-received was 19.8 which gradually decreased to 8.1 after 4^th^ pass ECAP of ZM21 Mg alloy. The basal planes are aligned ~45° to RD/TD which is favourable for the occurrence of basal slip because of the severe shear deformation; thus, a high Schmid factor of 0.35 for 4^th^ pass ECAP sample is obtained.
2. The ECAP process has substantial impacts on both the grain size and crystallographic orientation of ZM21 Mg. The greater the number of passes, yield and ultimate tensile strength decreased relatively. The % elongation of rolled ZM21 Mg enhanced from 20% to 27% at 4^th^ pass ECAP, nearly 26% increase was noticed.
3. After, 28 days immersion in Hank’s solution, % elongation of rolled and 4^th^ pass ZM21 Mg were reported to be 12.82 and 16.6%. This indicates ZM21 Mg alloys are suitable for surgical areas that requires high mechanical strength. Thus, ECAP successfully improved the mechanical integrity of magnesium implants.
4. The cell viability ratio of 99% indicated that 4^th^ pass ECAP ZM21 Mg are biocompatible in nature. Hence, 4^th^ pass ECAPed ZM21 are promising candidates for future in vivo studies.

## Supplementary information

Supplementary material
