# Supplementary material for: Bio-corrosion impacts on mechanical integrity of ZM21 Mg for orthopaedic implant application processed by equal channel angular pressing
Source: J Mater Sci Mater Med. 2021 Jun 12;32(6):65. doi: 10.1007/s10856-021-06535-5 (PMC8197699; doi:10.1007/s10856-021-06535-5)
Supplement: Supplementary file 1 — Supplementary material [file 10856_2021_6535_MOESM1_ESM.docx]

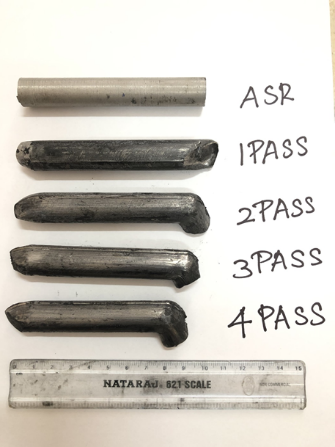


**Supplementary fig S1. Photograph of ECAPed sample.**


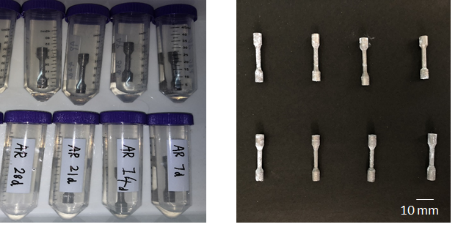


**Supplementary fig S2. Photograph of rolled and 4^th^ pass ZM21 Mg before and after 28 days corrosion in Hank’s solution**.
